# The Current Landscape of Antibiotic Use and Antimicrobial Resistance in Japan: Focusing on Common Infections Including Uncomplicated Urinary Tract Infection and Gonorrhea

**DOI:** 10.3390/antibiotics14080813

**Published:** 2025-08-08

**Authors:** Daisuke Fukuda, Yutaka Handa, Yoko Kayama, Kenji Fujii, Shinya Kawamatsu, Yoshiaki Kawano, Ivo Vojtek, Danielle Powell, Aruni Mulgirigama, Yoshiaki Gu

**Affiliations:** 1 Vaccine and Infectious Disease, GSK, Tokyo 107-0052, Japan; daisukefkd@gmail.com (D.F.);; 2 Specialty Care, GSK, Tokyo 107-0052, Japan; 3 Value Evidence and Outcomes, Specialty Care, GSK, Tokyo 107-0052, Japan; 4 Antibiotics, GSK, London WC1A 1DG, UK; 5 Department of Infectious Diseases, Center for Infectious Disease Education and Analysis (TCIDEA), Institute of Science Tokyo, Tokyo 113-8510, Japan

**Keywords:** antibiotic research, antimicrobial resistance (AMR), Japan, antibiotic development, national action plan

## Abstract

Antimicrobial resistance (AMR) has reached a critical situation globally, prompting urgent national responses to this escalating crisis, including the prioritization of novel antibiotic research. In 2016, Japan initiated a national AMR action plan that promoted appropriate antibiotic use in the country and encouraged a national environment conducive to mitigation measures. However, tackling AMR remains difficult. From an epidemiological perspective, this challenge now extends beyond severe infections, impacting common community-acquired infections, including uncomplicated urinary tract infections (uUTls) and gonorrhea. In uUTIs, the rising prevalence of extended-spectrum β-lactamase-producing and fluoroquinolone-resistant *Escherichia coli* diminishes the effectiveness of current, routinely used oral antibiotics, necessitating an exploration into innovative solutions. Similarly, the growing resistance of *Neisseria gonorrhoeae* to antibiotics such as azithromycin raises concerns about the efficacy of current therapeutic options for gonorrhea, which is a highly prevalent sexually transmitted infection. In Japan, since the removal of azithromycin as the recommended first-line treatment, there are no oral first-line antibiotics available to treat gonorrhea. Therefore, novel oral antibiotics are urgently needed for both serious and commonly occurring community-acquired infections. This narrative review discusses the limited availability of novel antibiotics in Japan, the distinctive features of the Japanese antibiotic repertoire and AMR epidemiology, and potential alternative oral treatments for community-acquired infections, including uUTIs and gonorrhea. Japan has been making significant advances toward tackling the AMR crisis through an updated national action plan, AMR policy changes, and innovative approaches to developing novel antibiotics. Substantial international cooperation and the engagement of diverse industry sectors are essential to address the pressing issue of AMR.

## 1. Introduction

Antimicrobial resistance (AMR) has emerged as a global health threat that exacerbates morbidity, mortality, and healthcare costs [1]. AMR places a significant strain on healthcare systems as available drugs become ineffective, making it harder to treat and contain infectious diseases that were previously quickly resolved [2,3]. AMR increases the likelihood of serious illnesses, uncontrolled disease spreading, and death [2,4]. The concern surrounding AMR is extending beyond severe or nosocomial infections, such as sepsis, pneumonia, complicated urinary tract infections (cUTIs), and pyelonephritis, to common community-acquired infections, such as uncomplicated urinary tract infections (uUTIs, also known as acute cystitis) and gonorrhea [5,6,7].

The global healthcare crisis of AMR is evident in countries like Japan, where the AMR Clinical Reference Center has reported increasing numbers of deaths due to fluoroquinolone-resistant *Escherichia coli* (*E. coli*)-derived bloodstream infections, with approximately 3000 deaths in 2015 and approximately 4000 deaths per year between 2018 and 2021 [8]. In Japan, AMR among community-acquired uUTIs has increased; for example, cefotaxime and levofloxacin resistance rates among *E. coli* isolates have increased from 8% and 25% in 2007 to 28% and 42% in 2017, respectively [9]. This rate of levofloxacin resistance in uUTIs from Japan in 2017 was higher than that reported globally by the World Health Organization (WHO) in their 2022 Global Antimicrobial Resistance and Use Surveillance System (GLASS) report [10]. This report outlined that, on average, approximately 38% of *E. coli* urinary tract infections (UTIs) were resistant at this time to levofloxacin across 45 countries, territories, and areas [10]. In addition, a nationwide surveillance study conducted in Japan between 2020 and 2021 reported a significant increase in the rate of fluoroquinolone-resistant and extended-spectrum β-lactamase (ESBL)-producing *E. coli* in uUTIs in post-menopausal women compared to a surveillance report from 2018 (fluoroquinolone-resistant *E. coli*: 20.7% vs. 32.1%, *p* = 0.0004; ESBL-producing *E.coli*: 10.0% vs. 15.4%, *p* = 0.0259) [11]. This rapid increase in resistance rates may be attributed to the excessive and persistent use of broad-spectrum oral antibiotics, including quinolones and cephem classes, within Japan [12]. Another example of the significant AMR threat in Japan is the increasing prevalence of multi-drug-resistant (MDR) *Neisseria gonorrhoeae* (*N. gonorrhoeae*) strains [13]. *N. gonorrhoeae* strains now have high documented levels of resistance to oral antibiotics previously used for gonorrhea treatment (fluoroquinolones, cefixime, and azithromycin), creating an unmet need for novel oral treatment options [13]. Parenteral ceftriaxone has retained its activity against *N. gonorrhoeae* and is the last remaining option for empirical first-line treatment [14], in part, through being able to be used at higher doses, especially through intravenous (IV) administration, in Japan if needed [15,16]. However, the first *N. gonorrhoeae* strain that is highly resistant to ceftriaxone was isolated in Japan in 2009, raising concerns for future treatment options [17,18].

The increasing incidence of AMR is particularly problematic in Japan because of the past and current landscape of antibiotic approval and use. Issues include a limited therapeutic repertoire, delays in or the lack of approval of novel antibiotics, and divergent treatment approaches, including the continued use of antibiotic classes for which rates of AMR are increasing at the community level [12,19].

This narrative review explores the evolving landscape of AMR in Japan through the lens of two urogenital infections: uUTIs, which are one of the most commonly occurring bacterial infections [20], and gonorrhea, due to the challenge it presents in terms of multi-drug resistance [21]. Several promising investigational oral antibiotic agents are discussed within. Information and data in this review were obtained by analyzing scientific journal articles sourced from PubMed searches, including late-phase clinical study results and pipeline analyses [1,22], plus internet sources like company press releases, clinical trial registers such as ClinicalTrials.gov, and biotechnology newsletters. We also searched the websites of the Pharmaceuticals and Medical Devices Agency (PMDA) [23], the US Food and Drug Administration (FDA) [24], the European Medicines Agency (EMA) [25], the WHO [26], and the Kyoto Encyclopedia of Genes and Genomes [27].

Please refer to the Supplementary Materials for a Plain Language Summary of this publication.

## 2. Current Unmet Antibiotic Needs in Japan

### 2.1. Inappropriate Use of Antibiotics

The proliferation of resistant bacteria resulting from inappropriate or suboptimal prescribing and/or use of antibiotics in community-acquired infections is of substantial concern in Japan [12]. A study analyzing outpatient antibiotic use between 2012 and 2015 found that prescription rates were particularly high in Japan, with approximately 704 antibiotic prescriptions per 1000 population per year compared with 506 per 1000 population in the US [12]. In addition, first-line antibiotics were prescribed in only approximately 24% of cases, and 70% of those prescribed for common infections were found to be inappropriately broad-spectrum, while more than 50% of antibiotic prescriptions were deemed to be unnecessary [12]. uUTIs are one of the most common reasons for antibiotic treatment in the community setting, accounting for approximately 33 antibiotic prescriptions per 1000 population per year in Japan [12]. Given the high frequency of uUTIs, appropriate antibiotic use is crucial to prevent the subsequent development of AMR [28]. Additionally, clinical isolates of AMR pathogens, such as ESBL-producing, fluoroquinolone-resistant, or carbapenem-resistant Gram-negative bacilli, have been increasingly reported in patients with uUTIs [29].

In line with US and European treatment guidelines, Japanese guidelines emphasize the critical need for promoting appropriate and optimal antibiotic use in order to prevent AMR, highlighting the need for a healthcare-wide strategy to monitor antibiotic use in uUTI management to preserve the efficacy of available treatments [30,31,32]. Recent Japanese Association for Infectious Disease (JAID)/Japanese Society of Chemotherapy (JSC) uUTI guidelines have advocated a tiered approach for appropriate antibiotic selection, designating amoxicillin/clavulanic acid as the first-choice antibiotic; cephem-class antibiotics are suggested for uUTIs caused by Gram-negative rods; and fluoroquinolones can be used for the treatment of Gram-positive or Gram-negative uropathogens [30]. Ultimately, the way to slow resistance rates in these broad-spectrum antibiotics is to control their use to an appropriate level [33]. A limited choice of alternatives may be a factor preventing this in Japan [29,34].

### 2.2. Novel Antibiotics for Community-Acquired Infections

Novel antibiotic development in Japan has primarily focused on addressing infections caused by highly resistant bacteria, such as carbapenem-resistant Enterobacterales (CREs), which can cause serious or life-threatening infections, including cUTIs, ventilator-associated bacterial pneumonia/community-acquired bacterial pneumonia, and complicated intra-abdominal infection [35,36,37,38,39,40,41,42,43,44,45,46,47,48,49,50,51,52,53,54,55,56,57,58,59,60,61,62,63,64,65]. This prioritization likely reflects the perceived urgency of life-threatening infections; however, the emergence of AMR is partly driven by the inappropriate use of antibiotics in common, community-acquired infections [66], and drug development efforts should be focused equally here. For example, the global increase in ESBL-producing and fluoroquinolone-resistant pathogens, coupled with the risk of highly MDR bacteria emerging from carbapenem use, presents an urgent need for the development of new antibiotics for uUTI treatment [11,67]. The uUTIs caused by MDR bacteria are not only difficult to treat, but recurrence can be a common and significant issue, and patients with a recurrent uUTI have an increased likelihood of AMR bacteria [68,69]. Furthermore, the presence of drug-resistant isolates in uUTIs increases the likelihood of progression to cUTI [70], which has an even higher reported prevalence of AMR pathogens [11,71] and higher healthcare utilization due to hospitalization [72]. Despite the clear importance of developing antibiotics for life-threatening infections, the significance of tackling AMR necessitates equal attention to community-acquired infections [73].

### 2.3. The Need for Novel Oral Antibiotic Options

While the global development of novel antibiotics remains limited, most approved therapies have consistently been IV options [74], such as imipenem/relebactam [51,52] and cefiderocol [59,62] (Table 1). While IV antibiotics are crucial, especially for critically ill patients, oral antibiotics offer distinct public health advantages in terms of accessibility; for example, they facilitate outpatient treatment, which can improve patient-related outcomes and minimize hospitalization needs (reducing healthcare costs and resource use) [75,76]. By reserving IV antibiotics for critical cases, oral antibiotics may contribute to optimizing healthcare infrastructure.

Gonorrhea is a significant public health concern in Japan, particularly among individuals between 25 and 35 years old [77]. While male patients often exhibit severe symptoms such as painful urethritis and discharge from the penis, female patients commonly present with subtler symptoms or remain asymptomatic [78], raising the risk of undiagnosed infections that not only increase the likelihood of spreading the disease but can subsequently lead to infertility [79]. The first-line treatment for gonorrhea in Japan is IV ceftriaxone, a third-generation cephem antibiotic, with spectinomycin, an aminoglycoside administered intramuscularly, serving as the secondary option [30]. The WHO and other health authorities worldwide have expressed growing concern over the emergence of MDR strains of *N. gonorrhoeae* [21,80]. A Japanese nationwide antibiotic susceptibility surveillance study published in 2023 (data collected in 2016) highlighted that, among the *N. gonorrhoeae* strains tested, all were resistant to penicillin G, 76.5% were resistant to ciprofloxacin, 47.1% were resistant to cefpodoxime, 18.8% were resistant to azithromycin, 8.2% were β-lactamase-producing strains, and 3.5% were resistant to cefixime [18]. None of those tested in the study were resistant to ceftriaxone or spectinomycin [18]; however, a ceftriaxone-resistant *N. gonorrhoeae* strain was identified in Japan in 2009 [81]. These resistance rates are generally higher than those reported globally by the WHO in their 2022 GLASS report, in which, on average, ~60% of *N. gonorrhoeae* isolates were resistant to ciprofloxacin, <10% were resistant to azithromycin and <3% were resistant to cefixime [10]. The limited number and variety of antibiotic options available to treat gonorrhea leads to a focusing of selection pressure, driving the emergence of resistant organisms against the few effective remaining antibiotics. Without the development of novel antibiotics, effective treatments for gonorrhea may become non-existent. The development of novel oral antibiotics may be preferential to IV/intramuscular antibiotics in the case of gonorrhea, as they would allow expedited partner therapy (the practice of treating the sexual partners of patients diagnosed with gonorrhea) to be easier to administer [82], enable outpatient treatment (reducing healthcare resource use) [83], and would also be useful for patients who are unwilling to receive IV or intramuscular medications.

**Table 1:** Year of approval, route of administration, and indications for recently approved (January 2015–June 2025) antibiotics by the FDA (US), EMA (EU), and PMDA (Japan).

	Antibiotic Class	US Approval Year	EU Approval Year	Japan Approval Year	Administration Forms	Indication	References
**Tedizolid**	Oxazolidinone	✓ (2014)	✓ (2015)	✓ (2018)	IV, PO	ABSSSI	[84,85,86,87]
**Oritavancin**	Lipoglycopeptide	✓ (2014)	✓ (2015)	✕	IV	ABSSSI	[88,89]
**Ceftolozane/** ** tazobactam**	BL/BLI combination	✓ (2014)	✓ (2015)	✓ (2019)	IV	cIAI, cUTI, HABP/VABP	[35,36,37,38]
**Ceftazidime/** ** avibactam**	BL/BLI combination	✓ (2015)	✓ (2016)	✓ (2024)	IV	cUTI, cIAI, HABP/VABP	[39,90]
**Rifaximin**	Rifamycin	✓ (2004)	✕	✓ (2016)	PO	Travelers’ diarrhea, overt hepatic encephalopathy, IBS-D	[85,91]
**Delafloxacin**	Fluoroquinolone	✓ (2017)	✓ (2019)	✕	IV, PO	ABSSSI, CAP	[40,41,92]
**Ozenoxacin**	Quinolone	✓ (2017)	✕	✓ (2015)	TOP	Impetigo, superficial skin infections, acne	[42,43]
**Secnidazole**	Nitroimidazole	✓ (2017)	✕	✕	PO	BV and trichomoniasis	[44]
**Meropenem/** ** vaborbactam**	BL/BLI combination	✓ (2017)	✓ (2018)	✕	IV	cUTI, cIAI, HABP/VABP	[39,45]
**Bedaquiline**	Diarylquinoline	✓ (2012)	✓ (2014)	✓ (2018)	PO	Pulmonary MDR-TB	[85,93,94]
**Fidaxomicin**	Macrolide	✓ (2011)	✓ (2011)	✓ (2018)	PO	Infectious enteritis caused by *C. difficile*	[85,95,96]
**Plazomicin**	Aminoglycoside	✓ (2018)	✕	✕	IV	cUTI	[46]
**Rifamycin**	Rifamycin	✓ (2018)	✕	✕	PO	Travelers’ diarrhea	[84,97]
**Eravacycline**	Tetracycline	✓ (2018)	✓ (2018)	✕	IV	cIAI	[47,48]
**Omadacycline**	Tetracycline	✓ (2018)	✕	✕	IV, PO	ABSSSI, CABP	[49]
**Sarecycline**	Tetracycline	✓ (2018)	✕	✕	PO	Acne	[50]
**Imipenem/** ** relebactam**	BL/BLI combination	✓ (2019)	✓ (2020)	✓ (2021)	IV	cUTI, cIAI, HABP/VABP	[51,52,98,99]
**Pretomanid**	Nitroimidazole	✓ (2019)	✓ (2020)	✕	PO	MDR-TB, pulmonary XDR-TB	[53,100]
**Lefamulin**	Pleuromutilin	✓ (2019)	✕	✕	IV, PO	CABP	[54]
**Lascufloxacin**	Fluoroquinolone	✕	✕	✓ (2019)	IV, PO	Respiratory tract and ENT infections; CABP	[55]
**Amikacin** ** liposome**	Aminoglycoside	✓ (2018)	✓ (2020)	✓ (2021)	Nebulizer dispersion	NTM lung infections caused by MAC	[56,57,58,101]
**Cefiderocol**	BL plus siderophore	✓ (2019)	✓ (2020)	✓ (2023)	IV	cUTI, HABP/VABP; Gram-negative infections with limited treatment options	[59,60,61,62]
**Durlobactam/** ** sulbactam**	BL/BLI combination	✓ (2023)	✕	✕	IV	HABP/VABP (*Acinetobacter baumannii-* * calcoaceticus* complex)	[63]
**Cefepime/** ** enmetazobactam**	BL/BLI combination	✓ (2024)	✓ (2024)	✕	IV	cUTI, HABP/VABP	[64,65,102]
**Sulopenem** ** etzadroxil**	Carbapenem	✓ (2024)	✕	✕	PO	uUTI	[103]
**Gepotidacin**	Triazaacenaphthylene	✓ (2025)	✕	✕	PO	uUTI	[103,104]
**Aztreonam/** ** avibactam**	BL/BLI combination	✓ (2025)	✓ (2024)	✕	IV	cIAI, cUTI, HABP/VABP	[105,106,107]

✓ = Approved for use; ✕ = Not approved for use; ABSSSI, acute bacterial skin and skin structure infections; BL, β-lactam; BLI, β-lactamase inhibitor; BV, bacterial vaginosis; CABP, community-acquired bacterial pneumonia; CAP, community-acquired pneumonia; cIAI, complicated intra-abdominal infections; cUTI, complicated urinary tract infection; ENT, ear, nose, and throat; EMA, European Medicines Agency; EU, European Union; FDA, US Food and Drug Administration; HABP/VABP, hospital/ventilator-associated bacterial pneumonia; IBS-D, irritable bowel syndrome with diarrhea; IV, intravenous; MAC, *Mycobacterium avium* complex; MDR-TB, multi-drug-resistant tuberculosis; NTM, non-tuberculous mycobacterial; PMDA, Pharmaceuticals and Medical Devices Agency; PO, oral administration; TOP, topical; US, United States; uUTI, uncomplicated urinary tract infection; XDR-TB, extensively drug-resistant tuberculosis.

## 3. Barriers to Diversifying Antibiotics in Japan

Based on the regulatory approvals of antibiotics between 2015 and 2025 (Table 1), it is apparent that the therapeutic repertoire is substantially smaller in Japan compared to other regions. There are several instances where antibiotics are approved in the US or Europe but not in Japan. Delafloxacin (formerly WQ-3034) serves as a pertinent example; originating from the Japan-based company Wakunaga Pharmaceuticals, this fluoroquinolone was approved in the US and Europe in 2017 and 2019, respectively, but has not yet been approved in Japan [40,41,92,108]. Another trend that can be visualized from the data in Table 1 is that even when antibiotics do gain approval in Japan, this often comes several years after approval in other territories. The US approval of cefiderocol, a collaborative discovery of Shionogi and GSK [109], occurred in 2019 [59,60]; however, the Japanese launch was delayed until late 2023 [62]. This issue is known as “drug lag”, whereby there is a difference in the duration of the new drug approval process between countries [19]. Drug lag is a major concern in Japan, with a median approval lag of 41 months compared with the US [19,110]. The delay or lack of novel antibiotic approvals in Japan is, in part, attributed to the distinctive business and regulatory landscapes governing antibiotic development [19]. Pharmaceutical companies often pursue concurrent drug development in the US and EU; similarities in the centralized drug approval process between the FDA and EMA mean that the integrated data package may be used for new drug applications in both regions within a similar timeframe [110]. However, drug approvals in Japan require a separate application to the Japanese PMDA and often require detailed local clinical data or additional/bridging clinical studies to be performed on Japanese patients, which can be costly [110,111]. Between 2016 and 2017, the EMA, FDA, and Japanese PMDA held meetings to harmonize drug approval processes across these regions and accelerate drug development to tackle AMR [112,113]. One of the key areas identified that required harmonization was requirements for clinical studies for specific types of infections, such as UTIs, and the agencies agreed to work to update guidance documents to reflect the agreed areas of convergence [113]. Further collaboration between regulatory agencies may help to increase the availability of novel antibiotic options in Japan. While misalignment in regulatory and approval processes between Japan and other countries may contribute to drug lag, delays in new drug applications for those of non-Japanese origin have also been highlighted [110]. Furthermore, when comparing the review times of new drug applications submitted in the US, EU, and Japan, the US review times were the shortest, and those in Japan were the longest [110]. While differences in review times were a factor in the approval delay in Japan, the late submission of new drug applications was considered a more critical factor in causing this lag [110]. In addition to late submissions and delayed review times contributing to Japan’s drug lag, in recent years, the pricing of novel antibiotics in Japan has also been consistently lower than in the US and Europe [114,115]. The reason for price differences between Japan and Western countries is unclear; however, this pricing misalignment has made the Japanese antibiotic market less attractive to the global pharmaceutical industry than other regions and undermines the reinvestment required to build a sustainable infectious disease therapeutic pipeline [116].

**Figure 1:**
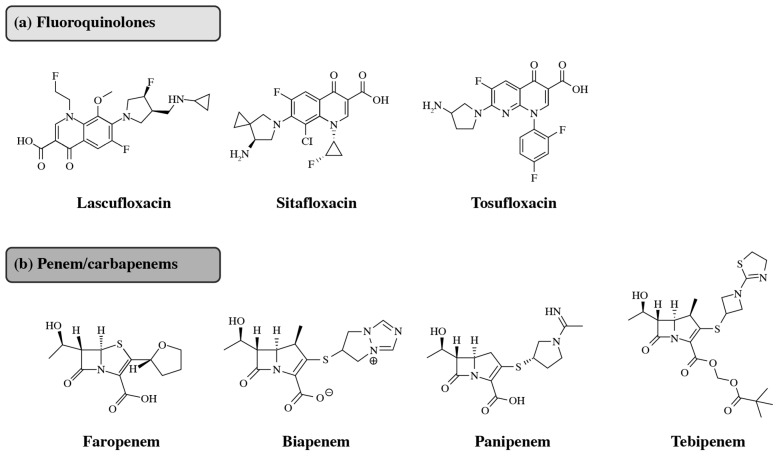
Antibiotics commonly used in Japan but not in the US or Europe. (**a**) Fluoroquinolones: lascufloxacin was launched by Kyorin Pharmaceutical in 2020 [55,85,129,134], sitafloxacin was launched by Daiichi Sankyo Company in 2008 [141], and tosufloxacin was launched by Toyama Chemical in 1990 [130,142]. (**b**) Penem/carbapenems: Faropenem was launched by Daiichi Sankyo Company in 1997 [143,144], biapenem was launched by Meiji Seika Pharma in 2002 [131], panipenem was launched by Daiichi Sankyo Company in 1993 [132,145], and tebipenem was launched by Meiji Seika Pharma in 2009 [146]. All were primarily introduced to the Japanese market. Figure 1 alt text: Chemical structures of seven antibiotics commonly used in Japan but not currently approved in the US or Europe. The figure is separated into two subfigures labeled (**a**) and (**b**). Part (**a**) displays the chemical structures of three fluoroquinolones: lascufloxacin, sitafloxacin, and tosufloxacin. Part (**b**) displays the chemical structures of four penem/carbapenems: faropenem, biapenem, panipenem, and tebipenem.

Japan has different treatment approaches when considering antibiotics compared with the US and Europe. For instance, trimethoprim/sulfamethoxazole, a key uUTI treatment in other countries, is reserved for the treatment of cUTIs in Japan [7,117]. Another example of alternative treatment approaches is in terms of dosage; amoxicillin/clavulanic acid is offered at multiple ratios in the US and Europe (2:1, 4:1, 7:1, 14:1, and 16:1), whereas Japan only offers the 2:1 ratio for administration in adults [118,119]. Nitrofurantoin is an oral antibiotic that is well-established for uUTI treatment in the US and Europe but is not currently an available treatment option for uUTIs in Japan [7,120]. Pivmecillinam, previously used in Japan, was withdrawn from the Japanese market but was approved in the US in April 2024 [29,121]. The market dominance of numerous fluoroquinolones and cephalosporins developed by Japan-based companies may be a factor contributing to the different approaches to antibiotic treatment in Japan [122,123,124,125]. Additionally, several antibiotics that are not approved in the US and Europe are approved for use in Japan (Figure 1) [55,126,127,128,129,130,131,132]. Lascufloxacin, sitafloxacin, and tosufloxacin are fluoroquinolones with potent antibacterial properties, predominantly available in Japan and other Asian countries (Figure 1) [55,133,134,135]. The antibiotic repertoire of a country contributes to the epidemiology of their AMR. For example, fluoroquinolones, a class of broad-spectrum antibiotics often used when first-line antibiotic therapy has failed due to AMR [136], are widely used in Japan (in particular levofloxacin), which has led to a distinct rise in the rate of fluoroquinolone-resistant bacteria within the country [33]. The FDA and EMA have included class labeling for fluoroquinolones, emphasizing that they should not be used for the treatment of uUTIs unless there are no other treatment options available [137,138]. This is due to concerns regarding adverse events linked to their use, reducing their risk–benefit ratio for use in less serious infections [138]. The broad use of fluoroquinolones has similarly been discouraged in the UK [139]. In Japan, a target for the reduced use of fluoroquinolones was set in the 2016 national action plan (discussed later in this review) and resulted in a decrease in their use [140]. However, specific instructions on how to reduce fluoroquinolone use have not been provided in Japan as they have in the US and the UK [137,138].

Several broad-spectrum β-lactam penem- and carbapenem-class antibiotics are approved for use in Japan (Figure 1) [131,132,143,144,146]. Faropenem, an oral penem-class antibiotic, is available mainly in Japan and India [147]. Additionally, several carbapenems, such as meropenem, were initially developed in Japan [148]. As with fluoroquinolones, carbapenems are a critical line of treatment in AMR [136]. Penem and carbapenem use is associated with CREs [149], and alongside those that produce ESBLs, CREs are on the list of the WHO’s priority pathogens [21,150]. Biapenem and panipenem are carbapenem-class antibiotics available mainly in Japan [151,152]. Tebipenem, an oral carbapenem, is currently accessible for pediatric use in Japan for the treatment of pneumonia, otitis media, and sinusitis [146].

A study investigating the link between antibiotic diversity and AMR in Gram-negative organisms in Japan found that antibiotic diversity is more important in preventing AMR than overall antibiotic use [153]. The limited diversity of available antibiotic classes in Japan creates selective pressure, increasing the risk of highly resistant bacteria [153].

## 4. AMR: Possible Solutions

### 4.1. National AMR Action Plan

An important aspect of tackling AMR is improving knowledge and providing guidance on AMR and the appropriate use of antibiotics to both the public and those engaged in the field of healthcare [33]. In 2015, the World Health Assembly adopted the Global Action Plan on Antimicrobial Resistance, calling on Member States to form multisectoral partnerships and create national AMR action plans based on a One Health approach [154]. The One Health approach aims to bring together stakeholders from all relevant sectors to design, implement, and monitor AMR programs, policies, legislation, and research in order to reduce AMR and achieve better health and economic outcomes [3]. National action plans were subsequently developed globally, including most countries in the Western Pacific Region [155].

Japan launched its first National Action Plan on Antimicrobial Resistance 2016–2020 (NAP1) in April 2016 [156]. NAP1 encompassed six comprehensive frameworks: (1) public awareness and education; (2) surveillance and monitoring; (3) infection prevention and control; (4) antimicrobial stewardship; (5) research and development (R&D); and (6) international cooperation, each with numerical targets set to be achieved by 2020 (which was extended to 2022 due to the COVID-19 pandemic) [33,140,156]. These frameworks largely aligned with key principles outlined in equivalent US and UK national action plans as well as the WHO global action plan [154], which included education and antimicrobial stewardship, monitoring and evaluation, infection control practices, international collaboration, and new antibiotic R&D [157,158]. Following the issue of the action plan, numerous studies on antimicrobial stewardship programs were published in Japan [159]. The Ministry of Health, Labour, and Welfare of Japan also subsequently published an antimicrobial stewardship manual for common infectious diseases in 2017 and 2019 [159,160]. NAP1 led to improved physician prescribing behaviors, resulting in an overall reduction in antibiotic use by 2020 [8,161]. A 2025 scoping review also found that antimicrobial stewardship programs established in Japan offered economic benefits in terms of drug and healthcare costs [162]. Despite these accomplishments, certain numerical outcome targets set out in the plan, such as the original goal of reducing the fluoroquinolone-resistance rate in *E. coli* strains to <25%, were not achieved (41.5% in 2020 vs. 38.0% in 2015) [29]. While it is difficult to directly compare the specific targets set by the Japan NAP1 to the US national action plan, both plans encompassed goals to reduce the rate of resistance for high-priority pathogens, the incidence of infections, and the over-use of antibiotics [156,157,158]. While the US national action plan did not set a specific target for the reduction in fluoroquinolone resistance, it set targets for 2020 to reduce carbapenem-resistant Enterobacterales infections acquired during hospitalization by 60%, MDR *Pseudomonas* spp. infections by 35%, and methicillin-resistant *Staphylococcus aureus* bloodstream infections by ≥50% versus 2011 values [33,157]. In the Japan NAP1, targets set for 2020 were to maintain the resistance level of *E. coli* at ≤0.2%, to lower the carbapenem (imipenem) resistance level of *Pseudomonas aeruginosa* to ≤10% (reported as 20% in 2014), and to lower the methicillin resistance level of *Staphylococcus aureus* to ≤20% (reported as 49% in 2014) [33]. When comparing the targets set by Japan and the US for 2020 versus 2015, the 13% reduction in fluoroquinolone resistance set by Japan was lower than the targets set for other resistance rates by the US national action plan [33,157]. While some of the targets set by the US national action plan were met, such as a 41% reduction in hospital-onset MDR *Pseudomonas aeruginosa* (versus the 35% target), others were not [163]. There was no significant reduction in the incidence of hospital-onset carbapenem-resistant Enterobacterales infections, and while there was a 31.5% decrease in hospital-onset methicillin-resistant *Staphylococcus aureus* bloodstream infections between 2012 and 2019, this did not meet the 50% target, and community-onset bloodstream infections remained stable [163]. The above observations highlight the need for further efforts to promote appropriate and optimal antibiotic use in Japan and globally, specifically with precious resources like fluoroquinolones and carbapenems that are needed to treat life-threatening infections [164,165]. Moreover, the simultaneous enhancement of novel antibiotic R&D is vital to expand the arsenal of antibacterial resources [166]. Despite the subsection on R&D within the Japan 2016 action plan, the initiative was not accompanied by an increase in R&D activity [33], as further demonstrated by the low number of drug approvals in Japan versus countries such as the US (Table 1).

Recent declines in antibiotic drug discovery and development in Japan can be attributed, in part, to the stagnation in Japan’s once prominent drug discovery expertise [33]. The acceleration of novel antibiotic creations can be achieved by prioritizing and resourcing R&D, including new natural resource exploration, microbial genome mining, mutasynthesis, and the advancement of novel screening methods [167,168,169,170]. Fostering innovation in antibiotic R&D was a key feature of the second national AMR plan in Japan (NAP2), published in 2023 (2023–2027) [33].

NAP2 was built on the framework of NAP1, retaining the same structure and six focus areas and emphasizing the WHO’s One Health approach to linking analysis and data for action across sectors and international findings [33]. NAP2 focuses on the introduction of financial incentives, including both public research funding support and market incentives, to support antibiotic research [33]. The public research fund support for R&D (often referred to as push incentives) includes the introduction of research support subsidies and tax benefits for R&D expenditures [33]. The Combating Antibiotic-Resistant Bacteria Biopharmaceutical Accelerator (CARB-X) and Biomedical Advanced Research and Development Authority (BARDA), both situated in the US, play a significant role in globally accelerating novel antibiotic development by providing funding and support in early development to combat drug-resistant bacteria [111]. Despite being US-based, the global funding of clinical trials in different populations and the network of collaboration supported by these organizations are vital to help bring the antibiotic pipeline to all patients. The Japan Agency for Medical Research and Development (AMED) takes a broader approach, spanning the wide spectrum of medical research, including novel antibiotics, to promote R&D [116]. Funding from institutions such as CARB-X, BARDA, and AMED plays a crucial role in advancing global antibiotic research efforts toward the discovery and development of innovative solutions [171,172].

Market-based financial incentives that motivate the promotion of R&D (known as pull incentives) were also included in the 2023 plan [33]. Examples of such pull incentives include the following: preferential post-market drug prices; the pre-screening of drug prices; the guaranteed purchase of new drugs; market entry incentives; a preferential exclusivity period; and the permission for the transfer of the exclusivity period to other products [33]. Similar incentive systems have been advocated by other countries, including the UK, Germany, and Sweden [173], prioritizing novel antibiotics with indications addressing the WHO’s priority pathogens [21,173]. The UK has recently piloted a subscription-style model whereby fixed payments for antibiotic access are set rather than linking reimbursement to the volume of drugs sold. The intended outcome of this model is that pharmaceutical companies will be encouraged to focus on novel antibiotic development through improved financial security [174]. If a similar model was developed in Japan, this may encourage pharmaceutical companies to enter the Japanese market with their novel antibiotics. The second national AMR action plan holds the potential to render the Japanese antibiotic market more attractive and stimulate the development of antibiotics to address AMR both now and in the future [33].

Key learnings from Japan’s experience in developing and implementing AMR national action plans, that could be useful to other countries, are the importance of integrating AMR efforts within broader health system strengthening initiatives and reinforcing antibiotic R&D and supply chains to ensure the availability of appropriate antibiotics [175]. Also, high-quality surveillance data are critical to estimating susceptibility trends, detecting emerging AMR, identifying new patterns of transmission, the early detection of outbreaks, monitoring response to interventions, and improving infection prevention and control [175]. Japan’s experience underscores the significance of contextual factors to the success of the national action plan. For instance, expanding AMR activities from hospitals to long-term care facilities has proven challenging in Japan due to differences in resources, workplace culture, and patient populations. It is important to understand a country’s specific situation before setting appropriate targets and planning how best to implement interventions. The critical success of a country’s national action plan also hinges on the collaborative efforts of the government, researchers, the pharmaceutical industry, and the broader healthcare community [116].

### 4.2. Resistance-Guided Therapy

While developing new antibiotic options is important in the fight against AMR, prolonging the use of those antibiotics already available through appropriate selection for treatment is equally valuable [176]. In support of this, the JAID/JSC guidelines advocate for urine culture and susceptibility testing not only for cUTIs but also for uUTIs to identify the presence of resistant bacteria to any relevant antibiotics [30]. The drawback of conducting urine culture and susceptibility testing is that it requires samples to be sent to the laboratory, and it can take several days for results to be obtained [177]. This means that patients with uUTIs must wait days from their initial consultation before receiving antibiotic treatment, during which they are likely to be in discomfort or pain [178]. Additionally, the process of susceptibility testing is argued to be more resource-intensive compared with empirical treatment [179]. However, if AMR is identified within patient infections prior to treatment, this enables the selection of the correct antibiotic to cure the infection the first time, thus reducing the requirement for additional doctor visits and antibiotic courses, improving patient health outcomes, slowing the rate of AMR, and reducing the financial burden on healthcare systems [180,181].

In 2015, a strategy known as resistance-guided therapy (RGT) was developed to reduce the empiric use of antibiotics with increasing resistance rates [182]. RGT involves the precise selection of first-line antibiotics for treatment based on the resistance profile of the bacteria causing each individual infection [182]. RGT aims to improve antimicrobial stewardship and the cure rate of first-line treatment [183]. In one previous study on *Mycoplasma genitalium* (*M. genitalium*), the empiric use of azithromycin was ceased and switched to an alternative antibiotic (doxycycline) while individualized testing was conducted to identify the macrolide resistance profile of the patient’s bacterial strain [183]. Following resistance profile results, treatment was adapted based on whether the strain was macrolide-resistant (in which case, azithromycin would not work, and so sitafloxacin was prescribed) or not (the treatment regimen was transferred to a higher dose of azithromycin) [182]. In a study conducted between 2016 and 2017, this RGT resulted in 95% and 92% cure rates in susceptible and resistant strains, respectively, and a reduction in selected macrolide resistance to less than 4% with a higher azithromycin dose [183,184]. Reducing therapy using a single recommended antibiotic (azithromycin in the case of *M. genitalium*) and treating conditions with different efficacious antibiotics (doxycycline/sitafloxacin for *M. genitalium*) may decrease the selection pressure for AMR and slow its continued emergence [185].

RGT has since been studied for its effectiveness in treating gonorrhea due to the increasing prevalence of MDR strains [185]. A study involving patients with *N. gonorrhoeae* infections was conducted whereby a polymerase chain reaction (PCR) assay was used to determine the resistance status of each individual to ciprofloxacin, an oral antibiotic treatment [185]. Patients with strains identified through RGT to be susceptible to ciprofloxacin were treated with a single dose of oral ciprofloxacin, with a 100% cure rate [185]. This suggests that the introduction of RGT for the treatment of gonorrhea infections could have substantial medical and public health benefits; however, this was only effective in those where gonococcal infections were determined to be susceptible to the alternate treatment option [185]. If a PCR assay determines that the infection is resistant to alternative antibiotics, then first-line treatment is the only option; as such, novel antibiotic options are still urgently required.

Despite the demonstrated effectiveness of RGT, this appears to have had limited adoption within Japan. A possible reason for the limited adoption of RGT could be due to the limited generation of antibiograms in healthcare facilities to support the appropriate selection of antibiotics [186]. Up-to-date susceptibility data and antibiograms in Japan, combined with education on how healthcare professionals can apply RGT for treatment selection, may allow more targeted and effective interventions to be designed and applied in the future. This is a key focus of NAP2 and will help to support the more appropriate prescribing of antibiotics [33]. Identifying the characteristics and circumstances of physicians who continue to prescribe antibiotics inappropriately will also be key to understanding and improving the current AMR situation in Japan [175]. Another possible reason for the limited implementation of RGT in Japan could be a lack of rapid susceptibility testing. The delay caused by urine culture and susceptibility testing in uUTIs often results in empiric treatment being preferable, which, in turn, contributes to the misuse of antibiotics and a rise in AMR [187]. The development of more rapid diagnostic tests may reduce patient and healthcare provider reliance on empiric prescribing, leading to improvements in patient care and antibiotic stewardship.

### 4.3. Novel Antibiotics in Development

**Figure 2:**
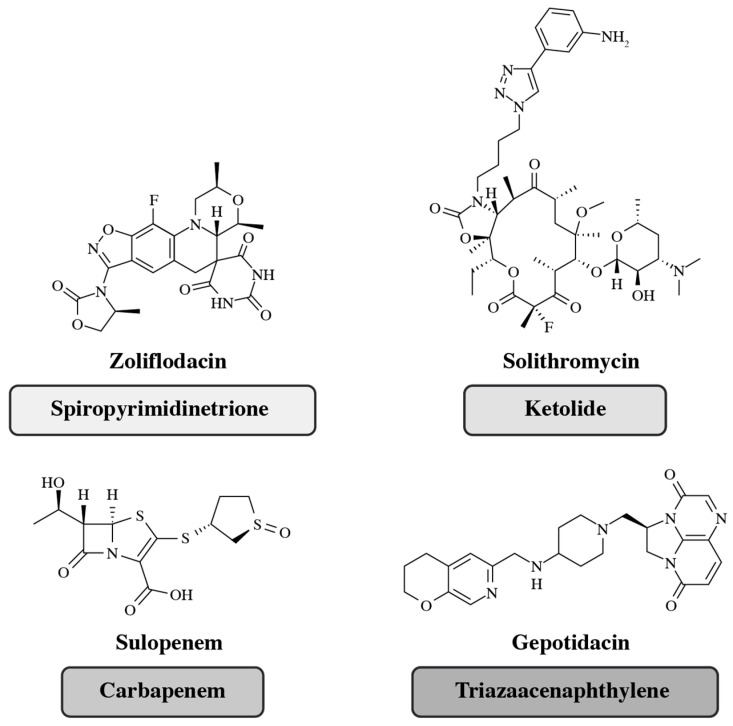
Investigational oral antibiotics that have advanced to Phase 3 clinical trials for the treatment of uUTIs and gonorrhea: zoliflodacin, solithromycin, sulopenem, and gepotidacin. Zoliflodacin and sulopenem (on the left) are being developed globally, excluding Japan. Sulopenem was recently approved for use in the US [103]. Solithromycin and gepotidacin (on the right) are being developed both globally and in Japan [166,189,191,192,193,194,195,196]. Figure 2 alt text: The chemical structures of four antibiotics that have advanced to Phase 3 clinical trials for the treatment of uncomplicated urinary tract infections and gonorrhea: zoliflodacin, solithromycin, sulopenem, and gepotidacin. The class of antibiotic is indicated in a box next to each chemical structure, which are spiropyrimidinetrione, ketolide, carbapenem, and triazaacenaphthylene, respectively.

The WHO has emphasized that the development of novel antibiotics is paramount in the fight against AMR [3], and it is encouraging to note that there are several new oral antibiotic candidates currently progressing through the global clinical pipeline. This section of the review highlights four antibiotics that have advanced to Phase 3 clinical trials, specifically tailored for the treatment of community-acquired urogenital infections such as uUTIs and gonorrhea: gepotidacin, solithromycin, sulopenem, and zoliflodacin (Figure 2) [188,189,190,191].

Gepotidacin is a first-in-class triazaacenaphthylene antibacterial that inhibits bacterial DNA replication via a unique mechanism of action (MoA), distinct binding site, and, for most pathogens, provides well-balanced inhibition of two different type II topoisomerase enzymes [197,198,199]. Due to the well-balanced inhibition of two enzymes, gepotidacin target-specific mutations in both enzymes are needed to significantly affect gepotidacin susceptibility [198,200]. This MoA provides in vitro activity against most strains of target uUTI uropathogens (such as *E. coli* and *Staphylococcus saprophyticus*) and *N. gonorrhoeae*, including bacterial isolates resistant to nitrofurantoin, fluoroquinolones, trimethoprim/sulfamethoxazole, and β-lactams [201,202,203]. The results from a Phase 3 trial of gepotidacin in patients with uncomplicated urogenital gonorrhea (EAGLE-1; NCT04010539) showed that oral gepotidacin demonstrated non-inferiority to intramuscular ceftriaxone and oral azithromycin combined therapy, which is the leading combination therapy for gonorrhea [188,204]. Two global Phase 3 trials comparing the efficacy and safety of oral gepotidacin to nitrofurantoin in female patients with uUTIs (EAGLE-2 [NCT04020341] and EAGLE-3 [NCT04187144]) both found gepotidacin to be non-inferior to nitrofurantoin, and the superiority of gepotidacin to nitrofurantoin was demonstrated in EAGLE-3 [196,205]. Based on the results of EAGLE-2 and EAGLE-3 [196], gepotidacin was approved in March 2025 by the FDA for the treatment of female adult and pediatric patients 12 years of age and older weighing at least 40 kg with uUTIs caused by the following susceptible microorganisms: *Escherichia coli*, *Klebsiella pneumoniae*, *Citrobacter freundii* complex, *Staphylococcus saprophyticus,* and *Enterococcus faecalis* [103,104]. A national Phase 3 study in Japan (EAGLE-J: NCT05630833) is underway to validate data in the Japanese patient population [193].

Solithromycin is an oral ketolide antibiotic undergoing clinical development for the treatment of community-acquired pneumonia and other infections [206]. This antibiotic has previously exhibited excellent in vitro activity against both Gram-positive and Gram-negative respiratory tract pathogens, including macrolide-resistant strains [207,208]. Moreover, solithromycin has demonstrated impressive in vitro antibacterial activity against MDR *N. gonorrhoeae* strains; yet, in a Phase 3 trial (NCT02210325) in patients with gonorrhea comparing solithromycin with ceftriaxone plus azithromycin, solithromycin failed to establish non-inferiority and had a higher frequency of adverse events [189]. Current efforts are focused on the development of solithromycin for community-acquired bacterial pneumonia and otorhinolaryngological infections, including ongoing development initiatives in Japan [195,209].

Sulopenem etzadroxil, the prodrug of IV sulopenem (CP-70429), is a promising new oral antibiotic derivative that is active against MDR Gram-negative pathogens, including those that produce ESBLs [190]. Sulopenem etzadroxil has been designed to enhance bioavailability and, when administered in combination with probenecid (a medication that increases uric acid excretion in the urine), has an extended half-life in plasma, prolonging antibacterial effects [190]. Combinations of sulopenem etzadroxil and probenecid have undergone Phase 3 clinical development and have since been approved for the treatment of uUTIs [190]. The Phase 3 clinical trial (NCT03354598) conducted on patients with uUTIs demonstrated that sulopenem was not non-inferior to ciprofloxacin in a ciprofloxacin-susceptible population [190]. Sulopenem etzadroxil and probenecid combination oral tablets have since been approved by the FDA for the treatment of uUTIs in 2024 [103].

Zoliflodacin (development codes: AZD0914 and ETX0914) is a first-in-class spiropyrimidinetrione being developed for the treatment of gonorrhea [194]. Zoliflodacin inhibits bacterial DNA replication through a unique MoA, whereby it inhibits bacterial type II topoisomerases through distinct binding sites compared with fluoroquinolones; fluoroquinolones bind to the GyrA subunit of DNA gyrase and the ParC subunit of DNA topoisomerase IV (topo IV), whereas zoliflodacin targets the GyrB (DNA gyrase) and ParE (DNA topo IV) subunits [194]. Zoliflodacin was shown to be non-inferior to ceftriaxone plus azithromycin in a Phase 3 trial (NCT03959527) [210,211]. Zoliflodacin underwent Phase 3 clinical development across five countries between 2019 and 2023, including Belgium, the Netherlands, South Africa, Thailand, and the US, but not Japan [191].

## 5. Concluding Remarks

Many antibiotics currently in use internationally first emerged between the 1950s and 2000s, and novel antibiotic development has slowed in recent times [212,213]. Programs to develop new antibiotics continue to be abandoned due to scientific challenges, regulatory issues, and limited commercial attractiveness [214]. Bacterial evolution in the face of therapeutic pressure leads to AMR, which drives a need for the considered use of existing resources and the constant development of new agents to keep pace with resistance [1]. Recognizing the gravity of the situation, a national AMR action plan was developed in Japan, emphasizing the appropriate use of antibiotics and incentivizing R&D investment. Despite this plan, Japan has witnessed few antibiotic developments, and these have mainly included IV compounds indicated for severe infections. The development of RGT as an option to make better use of currently available antibiotics has been a recent positive breakthrough. However, this appears to have had limited adoption in Japan; it is a concept reliant on the availability of alternative effective antibiotic options to first-line treatment, which, with AMR rates increasing as fast as they are presently, may soon no longer be available. Moving forward, improved rapid susceptibility testing methods, combined with RGT that utilizes novel oral antibacterial agents, are required to combat AMR. R&D into more rapid susceptibility testing for clinical practice could lead to the reduced empiric treatment of infections, improved adoption of RGT and, therefore, more successful treatment outcomes.

Of public health concern is the rise in AMR within commonly occurring community-acquired infections like uUTI and gonorrhea. The rising prevalence of fluoroquinolone-resistant *E. coli* in uUTIs indicates a serious challenge to antibiotic selection and has led to a rise in related fatalities such as bloodstream infections [8]. Recognizing how AMR in community infections can escalate to more serious conditions, there is a pressing need to stimulate drug discovery for novel antibiotics focused on combatting these more common infections. For gonorrhea, the first case of ceftriaxone-resistant *N. gonorrhoeae* was reported in Japan, and azithromycin-resistant *N. gonorrhoeae* are increasing globally, meaning that novel antibiotics for gonorrhea are needed [13]. This narrative review highlights the critical need for proactive strategies, including novel antibiotic development, to address the evolving landscape of AMR in Japan. Several promising investigational oral antibiotic agents are discussed within this review, signaling a proactive step towards combating AMR and ensuring a robust arsenal of new and effective antibiotics for future healthcare challenges.

Fostering collaborations between researchers, pharmaceutical industries, and regulatory bodies in Japan and other countries is crucial to the development of novel antibiotics for use in Japan. Global government action is also critical for the success of novel antibiotics and dealing with the current AMR crisis. As outlined in this review article, some key opportunities for action include: raising awareness of AMR; strengthening and implementing national AMR action plans and strategies; bolstering surveillance and monitoring; targeting infection prevention opportunities; promoting the responsible use of current antibiotics through antimicrobial stewardship/RGT; revitalizing antibiotic R&D with a restructured antimicrobial marketplace and improved marked-based financial incentives; an increased alignment between regulatory approval processes; and better engagement with other governments. It is also important for pharmaceutical companies to consider the inclusion of Japanese participants in Phase 3 clinical trials early in the development process. By learning from the experiences discussed in this review and embracing collaborative approaches, the Japanese community can work together to bridge the antibiotic development gap in Japan and effectively combat the growing threat of AMR.

## Data Availability

No new data were created or analyzed in this study.

## Supplementary Materials

The following supporting information can be downloaded at: https://www.mdpi.com/article/10.3390/antibiotics14080813/s1, Text: Lay Summary.
