# Supplementary material for: The Current Landscape of Antibiotic Use and Antimicrobial Resistance in Japan: Focusing on Common Infections Including Uncomplicated Urinary Tract Infection and Gonorrhea
Source: Antibiotics (Basel). 2025 Aug 8;14(8):813. doi: 10.3390/antibiotics14080813 (PMC12382829; doi:10.3390/antibiotics14080813)
Supplement: Supplementary file 1 [file antibiotics-14-00813-s001.zip › antibiotics-3637604-supplementary.pdf]

antibiotics

MDPI

Review

The Current Landscape of Antibiotic Use and Antimicrobial Resistance in Japan: Focusing on Common Infections Including Uncomplicated Urinary Tract Infection and Gonorrhea

Daisuke Fukuda[1], Yutaka Handa[2], Yoko Kayama[1], Kenji Fujii[1], Shinya Kawamatsu[3], Yoshiaki Kawano[3], Ivo Vojtek[1], Danielle Powell[4], Aruni Mulgirigama[4] and Yoshiaki Gu[5]

[1]Vaccine and Infectious Disease, GSK, Tokyo, Japan
[2]Specialty Care, GSK, Tokyo, Japan
[3]Value Evidence and Outcomes, Specialty Care, GSK, Tokyo, Japan
[4]Antibiotics, GSK, London, UK
[5]Department of Infectious Diseases, Center for Infectious Disease Education and Analysis (TCIDEA), Institute of Science Tokyo, Tokyo, Japan
*Correspondence: **author:** Danielle Powell, Antibiotics, GSK, 79 New Oxford Street, London, UK, WC1A 1DG. Email address: danielle.x.powell@gsk.com.

Lay summary:

- Bacteria are becoming increasingly resistant to currently available antibiotics, and this has reached a critical situation globally
- Antibiotic resistance impacts our ability to treat common community-acquired infectious diseases, such as urinary tract infections and gonorrhea, and puts a significant strain on the healthcare system
- Japan has distinctive challenges to overcome to tackle the current antibiotic resistance crisis, including a high level of inappropriate antibiotic prescriptions and a limited number of available antibiotics compared with other countries such as the United States
- New, oral, antibiotics effective against bacteria with resistance to current treatments are needed for both serious and commonly occurring community-acquired infections in Japan
- Japan has been making significant advances towards tackling the antibiotic resistance crisis through an updated national action plan, policy changes, and innovative approaches to developing novel antibiotics
- Potential new oral antibiotics for the treatment of urinary tract infections and gonorrhea include zoliflodacin, solithromycin, sulopenem, and gepotidacin
- Fostering collaborations between researchers, pharmaceutical industries, and regulatory bodies in Japan and other countries is crucial to developing new antibiotics in Japan and effectively combating the growing threat of antibiotic resistance
